# Features Associated With Depressive Predominant Polarity and Early Illness Onset in Patients With Bipolar Disorder

**DOI:** 10.3389/fpsyt.2020.584501

**Published:** 2020-11-16

**Authors:** Jesús García-Jiménez, Luis Gutiérrez-Rojas, Sara Jiménez-Fernández, Pablo José González-Domenech, María D. Carretero, Manuel Gurpegui

**Affiliations:** ^1^Southern Mental Health Clinical Management Unit, Santa Ana Hospital, Motril, Spain; ^2^Department of Psychiatry, University of Granada, Granada, Spain; ^3^Psychiatry and Neurosciences Research Group (CTS-549), Institute of Neurosciences, University of Granada, Granada, Spain; ^4^Granada Mental Health Clinical Management Unit, Hospital Clínico San Cecilio, Granada, Spain; ^5^Child and Adolescent Mental Health Service, Jaén University Hospital Complex, Jaén, Spain

**Keywords:** bipolar disorder, age at onset, disability, polarity predominance, diagnosis delay

## Abstract

**Objective:** The aim of this study is to determine the prevalence of three possible diagnostic specifiers, namely predominant polarity (PP) throughout illness, polarity of the first episode and early age at onset, in a sample of bipolar disorder (BD) patients and their association with important socio-demographic, clinical and course-of-illness variables.

**Methods:** A retrospective and naturalistic study on 108 BD outpatients, who were classified according to the PP, polarity of the first episode and early age at onset (≤ 20 years) [vs. late (>20 years)] and were characterized by their demographics, clinical data, functionality and social support, among others features. After bivariate analyses, those variables showing certain association (*P* value < 0.25) with the three dependent variables were entered in logistic regression backward selection procedures to identify the variables independently associated with the PP, polarity of the first episode and early age at onset.

**Results:** The sample consisted of 75 women ad 33 men, 74% with type I BD and 26% with type II. Around 70% had depressive PP, onset with a depressive episode and onset after age 20. Depressive PP was independently associated with depressive onset, higher score on the CGI severity scale and work disability. Onset with depressive episode was associated with type II BD, longer diagnostic delay and higher score on family disability. Early age at onset (≤ 20 years) was associate with younger age, longer diagnostic delay, presence of ever psychotic symptoms, current use of antipsychotic drugs and higher social support score.

**Conclusions:** The results of this study show that BD patients with depressive PP, onset with depression and early age at onset may represent greater severity, because they are frequently associated with variables that worsen the prognosis. Our findings match up with the conclusions of two systematic reviews and we also include a disability factor (at family and work) that has not been previously reported. This work contributes to the use of polarity and age at onset in BD patients, as it can become a useful instrument in the prognostic and therapeutic applications.

Bipolar disorder (BD) is a chronic mood disorder with a global prevalence of 1–5% (1, 2), which is associated with high comorbidity rates, a large number of premature deaths due to suicide and a worse social and work performance (3–5). All of those characteristics entail a significant economic impact due to both direct and indirect costs (1) and require an effective diagnostic and therapeutic approach.

Diagnostic manuals [the International Statistical Classification of Diseases and Related Health Problems 10th Revision (ICD-10) (6) and the Diagnostic and Statistical Manual of Mental Disorders (DSM-5) (7)] compile the diagnostic criteria to correctly diagnose each of the episodes appearing in BD (mania, hypomania, and depression). The DSM-5 also proposes the use of specifiers for BD and related disorders, called Diagnostic Specifiers (DS) (7). The use of these DS adds relevant information on factors of poor prognosis of the acute relapse, allows focusing on the most conflicting points and, therefore, it leads to a more individualized approach to BD. Currently, the DSM-5 has accepted the use of nine DS for BD (7). For example, the DS “anxious distress” is associated with a higher risk of suicide, longer acute episodes and a worse response to treatment. Thus, a patient with an acute decompensation who also meets the criteria of “anxious distress” will present a risk of suicidal behavior significantly higher than another patient without this DS and, therefore, the treatment of this patient should especially consider this circumstance.

Several concepts related to BD have been proposed as DS in recent years, such as insight or response to lithium, but our study focuses on two of them, the age of onset (AO) and the polarity [polarity at onset (OP)and predominant polarity(PP)]. In the literature, many original articles conclude that AO, OP, and PP are associated with important prognostic factors (see below). However, two systematic reviews recently published indicate the existence of some contradictory associations, which could have influenced that they were not finally included in the DS list of the DSM-5 (8, 9). Regarding the AO, the meta-analysis of Joslyn et al. (9) indicates that patients under 20 years of age at BD onset show more frequently a greater diagnostic delay (10–12), longer delay in treatment initiation (9–14), they have more severe depressive symptoms (10–12, 15), high comorbidity with anxiety disorders (15–17) and higher risk of substance abuse (18, 19), all of which are associated with worse clinical course. The authors of this meta-analysis also report that the methodological variability and criteria when establishing a cut-off point for AO was very high among the selected studies and it could be a determining factor when no statistical association was found with variables that classically have also been related to a poor prognosis and early AO, such as psychotic symptoms (20) or suicidal risk (21).

The PP, defined as the tendency to present more frequently manic or depressive decompensations, and the OP or type of polarity of the index episode have been studied in another systematic review (8). The results of this study suggest that patients who are usually decompensated to the manic pole, i.e., manic PP (MPP), usually initiates with a manic episode (22–24), respond better to atypical antipsychotics and mood stabilizers (25) and they are frequently consumers of toxic substances prior to the onset of BD (24, 26, 27). Regarding depressive PP (DPP) patients, the most frequent initial episode found is of depressive characteristics (22, 28) and they present more suicidal risk (24, 26) and comorbidity with anxiety disorders (29). DPP also seems to be associated with a greater diagnostic delay (22, 28) and it has a characteristic pharmacological profile due to a greater use of quetiapine and lamotrigine (25). Again, the need to unify methodology and criteria in the study of the PP is pointed out, because contradictory results were also detected despite all these associations. These inconsistencies must be clarified before being able to recommend the systematic use of polarity when approaching the study of BD (8).

We conducted a study on a sample of outpatients diagnosed with BD. The main objective was to describe the prevalence of OP, PP, and AO. The second objective was to analyze the sociodemographic, clinical and therapeutic characteristics that are significantly associated with the three main variables of the study. Thus, we aim to analyze the usefulness of OP, PP, and AO as DS contributing to individualize and, therefore, optimize the clinical approach of BD.

## Methods

### Participants

The methodology used has been previously described by Gutiérrez-Rojas et al. (30). In summary, a psychiatrist (LGR) collected data from BD patients receiving treatment at an outpatient mental health clinic in the city of Jaén (Spain). A total of 210 patients were identified as suffering from BD. Out of these 210 patients, four had died (three by suicide), and 35 could not be located; of the remaining 171 patients, 16 (9%) refused to participate, nine fulfilled the exclusion criteria (see below), and 38 did not fulfill the diagnostic criteria. The remaining 108 patients were interviewed as they were attending the outpatient clinics or by specific appointment, after providing their informed consent to participate in the study. The patients were specifically interviewed for this research purpose in a personal appointment. They usually went to the clinic with their principal caregiver. The protocol was approved by the Ethics Committee of the Jaén Hospital Complex. Patients under 18 years of age and patients affected by a severe cognitive deficit, as dementia or intellectual disability, were excluded.

### Assessment Instruments and Procedure

Axis I psychopathology was measured in each interview using the Structured Clinical Interview for DSM-IV (SCID) (31). The referring psychiatrist was asked to confirm the diagnosis (according to the same criteria) and the therapeutic regimen.

The study has a retrospective design. Several data (for example the presence of affective symptoms, level of disability or social support) was measured in the initial interview meanwhile prognosis variables (as number and kind of episodes, PP, OP, AO and family history of mental disorders) were analyzed using the information from the medical records and from the interview with the patients and their principal caregivers. The following information was collected in the initial interview: sociodemographic data (sex, age, marital status, number of siblings and children, educational level and work status), clinical variables (AO of symptoms, age at diagnosis of BD, years of illness, OP [depressive, (hypo) manic or mixed], number of episodes of each polarity, number of hospital admissions and suicide attempts). Diagnosis delay was defined as the time (in years) between the first appearance of affective symptoms and the correct diagnoses of BD. The OP refers to first mood episode that the patient suffers and not to the index episode of consultation. The AO of symptoms was defined as the age at which the first manic episode or major depressive episode occurred and was classified as early (≤ 20 years) or late (>20 years). The PP was defined by the higher number of episodes of one subtype (manic) or the other (depressive), as previous studies have used (28). The principal reasons to adopt this definition are the relatively small sample size, the cross-sectional nature of the assessments, and the retrospective design. If we had chosen to divide the sample in three groups (with the option of indeterminate PP) we would not get enough statistical power.

The affective symptomatology was evaluated using the Young's scale for manic symptoms –YMRS- (32) and the Hamilton's scale for depression –HDRS- (33). Due to the naturalistic design of the study, we included euthymic and non-euthymic patients. Moreover, we administered the Clinical Global Impression (CGI) severity scale (34).

Social support was measured by Social Adaptation Self-evaluation Scale (SASS) (35) which is made up of 21 items that explore the functioning of the patient in five areas: work, family, leisure, social relationships, and motivational skills. The score ranges between 0 and 60 (a higher score means better social support).

To evaluate the substance abuse of legal (tobacco and alcohol) and illegal substances we used the Fagerström Test for Nicotine Dependence (FTND) (36) and the CAGE questionnaire (37). To measure the three principal dimensions of the disability (work, social and family) we used the Sheehan Disability Scale (SDS) (38). This scale is a brief self-rated assessment of impairment caused by a disease in three dimensions: work or studies, social life (and leisure) and family life (home responsibility) during the last week. Each item has 11 possible responses ranging from 0 through 10 (0, not at all; 1–3, mildly; 4–6, moderately; 7–9, markedly; and 10, extremely). The psychometric properties of SDS have been previously evaluated in BD (39) and have been validated in Spanish (40). Significant functional impairment for work as well as for social life and family life has been defined as a score of 7 or higher in patients with a positive screen for BD (41).

### Statistical Analysis

Collected data were summarized using descriptive measures (mean, median, standard deviation, frequency, and percentage). Continuous variables were analyzed using the Student's *t*-test or analysis of variance (ANOVA) and categorical variables were determined using the Pearson's chi-square test or the Fisher's exact test. For the bivariate analysis, as cautious approach to multiple testing, rather than using Bonferroni correction, we have set two-tail statistical significance was set at *P* < 0.01. The analyses were performed using the Statistical Package for the Social Sciences (SPSS) program (version 24).

Differences between groups were analyzed using standard bivariate parametric or nonparametric tests as appropriate, comparing sociodemographic variables, the subtype of BD, the presence of psychotic symptoms at some point throughout the disease, current or past substance abuse, delay in diagnosis delay, types of psychopharmacological treatments, illness duration, social support and disability extent. These variables were entered in the logistic regression backward selection procedures examining their association with dependent outcomes of interest (PP, OP and early AO of BD) when their bivariate association reached a *P* value < 0.25, in order to adjust for potential confounding variables. All logistic regression models fit well according to the Hosmer-Lemeshow goodness of fit test (42).

## Results

The sample included 108 participants (33 men and 75 women) with a median age of 48 ± 14 years. A total of 80 (out of 108, 74.1%) participants met the type I BD criteria and 28 (25.9%) participants were diagnosed with type II BD. Regarding the PP, 29.6% of the sample showed MPP compared to the 70.4% that showed DPP. Table 1 summarizes the main sociodemographic, clinical and course-of-illness variables related with the PP. The depressive PP group (in comparison with the manic PP) showed significantly higher proportion of patients with type I BD (vs. type II), more diagnostic delay, higher score on the CGI severity scale, higher number of depressive episodes, more frequently onset with depressive episode and lower proportion of patients with ever psychiatric hospitalization.

**Table 1:** Predominant polarity and their associated variables.

	**Manic predominance [32 (29.6%)]**	**Depressive predominance [76 (70.4%)]**	**Statistic, *P***
Age, mean (SD)	46.9 (11.4)	48.7(15.1)	−0.68^a^, 0.50
Sex [*n* (%)]			0.01^b^, 0.92
Male	10 (30.3)	23 (69.7)	
Female	22 (29.3)	53 (70.7)	
Marital status [*n* (%)]			0.10^b^, 0.75
Currently married	20 (62.5)	45 (59.2)	
Currently unmarried	12 (37.5)	31 (40.8)	
Educational level [*n* (%)]			0.44^c^, 0.51
Primary school	17 (53.1)	44 (57.9)	
Secondary school	8 (25)	20 (26.3)	
University	7 (21.9)	12 (15.8)	
Work status [*n* (%)]			2.07^c^, 0.15
Disabled (temporarily/permanent)	20 (62.5)	31 (40.8)	
Unemployed	3 (9.4)	20 (26.3)	
Working (full/part-time)	9 (28.1)	12 (15.8)	
Diagnostic category [*n* (%)]			9.17^b^, **0.002**
Bipolar disorder I	30 (37.5)	50 (62.5)	
Bipolar disorder II	2 (7.1)	26 (92.9)	
Delay in diagnosis in years, mean (SD)	5.5 (6.4)	10.2 (10.0)	−2.92^a^, **0.004**
Ever psychotic symptoms [*n* (%)]	15 (46.9)	25 (32.9)	1.89^b^, 0.17
Ever psychiatric hospitalization [*n* (%)]	28 (87.5)	45 (41.7)	8.23^b^, **0.004**
Ever suicide attempt [*n* (%)]	9 (28.1)	29 (38.2)	0.99^b^, 0.32
Current treatment [*n* (%)]			
Mood stabilizers	26 (81.2)	66 (86.8)	0.56^b^, 0.46
Antipsychotics	17 (53.1)	31 (40.8)	1.39^b^, 0.24
Antidepressants	6 (18.8)	34 (44.7)	6.52^b^, 0.011
Benzodiazepines	16 (50)	41 (53.9)	0.14^b^, 0.71
CGI score, mean (SD)	2.6 (1.6)	3.5 (1.5)	−2.68^a^, **0.009**
Age at onset [*n* (%)]			1.31^b^, 0.25
≤ 20 years	7 (21.9)	25 (32.9)	
>20 years	25 (78.1)	51 (67.1)	
Family history of psychiatric illness [*n* (%)]	22 (68.8)	54 (71.1)	0.04^b^, 0.81
Polarity at onset [*n* (%)]			19.18^b^, < **0.001**
Manic or mixed	17 (53.1)	10 (13.2)	
Depressive	15 (46.9)	66 (86.8)	
Work disabilityscore, mean (SD)	7.8 (3.3)	7.4 (3.6)	0.49^a^, 0.62
Social disability score, mean (SD)	5.3 (3.8)	5.9 (3.6)	−0.66^a^, 0.51
Family disability score, mean (SD)	4.8 (4.1)	6.3 (3.5)	−1.71^a^, 0.093
Total number of episodes, mean (SD)	10.2 (7.5)	10.7 (10.2)	−0.28^a^, 0.78
Number of manic episodes, mean (SD)	7.1 (6.0)	2.8 (2.3)	3.73^a^, **0.001**
Number of depressive episodes, mean (SD)	3.3 (2.9)	5.8 (4.1)	−3.05^a^, **0.003**
SASS social support score, mean (SD)	36.5 (9.1)	33.9 (10.6)	1.22^a^, 0.23
Alcohol misuse [*n* (%)]			0.07^b^, 0.79
CAGE <2	28 (87.5)	65 (85.5)	
CAGE ≥ 2	4 (12.5)	11 (14.5)	
Nicotine dependence [*n* (%)]			0.24^b^, 0.63
FTND <6	23 (71.9)	58 (76)	
FTND ≥ 6	9 (28.1)	18 (24)	

CGI, clinical global impression; SASS, social adaptation self-evaluation scale; CAGE, cut-down, annoyed, guilty, eye-opener (questionnaire); FTND, Fagerström Test for nicotine dependence; SD, standard deviation.

a Student's t test.

b Chi-square test.

c ANOVA.

*Bold value indicates significance level inferior to 0.01*.

Regarding the onset polarity, a depressive beginning of the illness appeared in 75% of patients (25% started with a manic episode). The age at onset in the sample had a mean of 30.4 (SD, 13.7) years. Early onset (≤ 20 years) was present in 30% of the patients [late onset (>20 years) in 70%]. Onset with manic polarity showed the same proportion (25%) among those with early onset than among those with late onset. Table 2 shows the results of the bivariate analysis of variables according to early or late age at onset. The factors associated with an early age at onset were: younger age, higher proportion of unmarried marital status, ever use of antipsychotic drugs and larger diagnosis delay.

**Table 2:** Association of variables with early or late onset in bipolar disorder.

	**Early age at onset (≤20 years) (32, 29.6%)**	**Late age at onset (>20 years) (76, 70.4%)**	**Statistic, *P***
Age, mean (SD)	39.2 (13.1)	52.0 (12.8)	4.68^a^, ** < 0.001**
Sex [*n* (%)]			2.17^b^, 0.14
Male	13 (39.4)	20 (60.9)	
Female	19 (25.3)	56 (74.7)	
Marital status [*n* (%)]			9.77^b^, **0.002**
Currently married	12 (37.5)	53 (69.7)	
Currently unmarried	20 (62.5)	23 (31.3)	
Educational level [*n* (%)]			0.02^c^, 0.88
Primary school	17 (53.1)	44 (57.9)	
Secondary school	11 (34.4)	17 (22.4)	
University	4 (12.5)	15 (19.7)	
Work status [*n* (%)]			0.50^c^, 0.48
Disabled (temporarily/permanent)	16 (75)	35 (46.1)	
Unemployed	8 (25)	15 (19.7)	
Working (full/part-time)	8 (25)	26 (34.2)	
Diagnostic category [*n* (%)]			1.22^b^, 0.27
Bipolar disorder I	26 (32.5)	54 (67.5)	
Bipolar disorder II	6 (21.4)	22 (78.6)	
Delay in diagnosis in years, mean (SD)	13.9 (12.3)	7.1 (7.2)	−2.52^a^, 0.016
Ever psychotic symptoms [*n* (%)]	15 (46.9)	25 (32.9)	1.89^b^, 0.17
Ever psychiatric hospitalization [*n* (%)]	24 (75)	49 (64.5)	1.14^b^, 0.29
Ever suicide attempt [*n* (%)]	15 (46.9)	23 (30.3)	2.73^b^, 0.099
Current treatment [*n* (%)]			
Mood stabilizers	27 (84.4)	65 (85.5)	0.02^b^, 0.88
Antipsychotics	21 (65.6)	27 (35.5)	8.26^b^, **0.004**
Antidepressants	10 (31.2)	30 (39.5)	0.65^b^, 0.42
Benzodiazepines	15 (46.9)	42 (55.3)	0.64^b^, 0.43
CGI score, mean (SD)	3.4 (1.7)	3.1 (1.5)	−1.0^a^, 0.32
Polarity predominance [*n* (%)]			1.31^b^, 0.25
Manic	7 (21.9)	25 (32.9)	
Depressive	25 (78.1)	51 (67.1)	
Family history of psychiatric illness [*n* (%)]	23 (71.9)	53 (69.7)	0.049^b^, 0.824
Polarity at onset [*n* (%)]			0.00^b^, 1.00
Manic or mixed	8 (25)	19 (25)	
Depressive	24 (75)	57 (75)	
Work disability score, mean (SD)	8.1 (3.2)	7.3 (3.6)	−1.04^a^, 0.30
Social disability score, mean (SD)	6.5 (3.3)	5.4 (3.8)	−1.50^a^, 0.14
Family disability score, mean (SD)	5.7 (3.8)	5.9 (3.7)	0.28^a^, 0.78
Total number of episodes, mean (SD)	14.1 (10.6)	8.9 (8.0)	−2.38^a^, 0.020
Number of manic episodes, mean (SD)	5.9 (6.7)	4.2 (3.7)	−1.44^a^, 0.15
Number of depressive episodes, mean (SD)	6.8 (4.6)	3.9 (3.1)	−2.73^a^, 0.011
SASS social support score, mean (SD)	34.8 (10.0)	34.6 (10.4)	−0.10^a^, 0.92
Alcohol misuse [*n* (%)]			4.69^b^, 0.030
CAGE <2	24 (75)	69 (90.8)	
CAGE ≥ 2	8 (25)	7 (9.2)	
Nicotine dependence [*n* (%)]			5.73^b^, 0.017
FTND <6	19 (59.4)	61 (81.3)	
FTND ≥ 6	13 (40.6)	14 (18.7)	

CGI, clinical global impression; SASS, social adaptation self-evaluation scale; CAGE, cut-down, annoyed, guilty, eye-opener (questionnaire); FTND, Fagerström Test for nicotine dependence; SD, standard deviation.

a Student's t test.

b Chi-square test.

c ANOVA.

*Bold value indicates significance level inferior to 0.01*.

Multivariate logistic regression analyses were performed to identify the variables significantly associated with depressive PP, depressive polarity of the first episode and early age at onset (Tables 3–5, respectively). The factors associated with depressive PP (Table 3) were the following: being work disabled due to the BD, having had depressive onset and higher score on the CGI severity scale (the use of antidepressant medication was significant in the bivariate but not in the multivariate analysis). Variables associated with an onset depressive polarity (Table 4) were type II BD, higher family disability (rated on the SDS score) and a longer diagnostic delay. Finally, early age at onset was significantly associated with younger age, longer diagnostic delay, higher social support score, presence of ever psychotic symptoms and current use of antipsychotic drugs (Table 5).

**Table 3:** Logistic regression of factors associated with a depressive predominant polarity in a bipolar disorder sample (*n* = 108).

**Variables**	***n***	**%**	**Crude OR**	**95% CI**	**Pearson** **χ^2^**	**df**	***P* value**	**Adjusted OR**	**95% CI**	**Wald** **χ^2^**	**df**	***P* value**
Polarity of first episode					19.18	1	** <0.001**			14.84	1	** <0.001**
Manic or mixed	10/27	37	1.0					1.0				
Depressive	66/81	81	7.5	(2.9–19.6)				8.9	(2.9–27.1)			
Work disabled due to disorder					3.45	1	0.063			5.26	1	**0.022**
Yes	35/56	62	1.0					1.0				
No	41/52	79	2.2	(0.95–5.3)				3.5	(1.2–10.2)			
Use of antidepressant					6.52	1	**0.011**			3.41	1	0.065
No	42/68	62	1.0					1.0				
Yes	34/40	85	3.51	(1.29–9.5)				2.9	(0.9–9.2)			
	**Mean**	**SD**		**Student's** ***t***	**df**	***P*** **value**	**Adjusted OR**	**95% CI**	**Wald** **χ^2^**	**df**	***P*** **value**	
CGI severity score				2.68	106	**0.009**	1.6	(1.2–2.3)	8.22	1	**0.004**	
In patients with manic PP	2.6	1.6										
In patients with depressive PP	3.5	1.5										

*In addition to the variables showed in the table, the variables initially included in the logistic regression backward selection procedure were sex, age, age at onset of bipolar disorder, subtype of bipolar disorder, history of psychotic symptoms, diagnosis delay (in years), subtype of psychopharmacological treatment, hospitalization ever, Clinical Global Impression score and social support score. For the logistic regression, the Hosmer-Lemeshow goodness-of-fit test showed that the logistic model was appropriate (χ^2^ = 7.15; df = 7; P = 0.41). Bold value indicates significance level inferior to 0.01*.

**Table 4:** Logistic regression of factors associated with depressive polarity of the first episode in a bipolar disorder sample (*n* = 108).

**Variables**	***n***	**%**	**Crude OR**	**95% CI**	**Pearson** **χ^2^**	**df**	***P* value**	**Adjusted OR**	**95% CI**	**Wald** **χ^2^**	**df**	***P* value**
Predominant polarity					19.18	1	** <0.001**			7.19	1	**0.007**
Manic or mixed	15/32	46	1.0					1.0				
Depressive	66/76	87	7.5	(2.9–19.6)				5.1	(1.5–16.7)			
Subtype of bipolar disorder					9.26	1	**0.002**			4.34	1	**0.037**
Type I	54/80	67	1.0					1.0				
Type II	27/28	96	13.0	(1.7–100.9)				10.2	(1.1–90.8)			
Family disability score					2.79	1	0.095			5.15	1	**0.023**
Less than 7 (well)	42/51	82	1.0					1.0				
7 or more (poor)	39/57	53	1.4	(0.98–1.9)				4.1	(1.2–13.9)			
	**Mean**	**SD**		**Student's** ***t***	**df**	***P*** **value**	**Adjusted OR**	**95% CI**	**Wald** **χ^2^**	**df**	***P*** **value**	
Diagnosis delay (in years)				−5.84	105	** <0.001**	1.24	(1.09–1.4)	9.96	1	**0.002**	
Manic or mixed first episode	3.1	3.6										
Depressive first episode	10.7	9.9										

*In addition to the variables showed in the table, the variables initially included in the backward logistic regression backward selection procedure were sex, age at onset of bipolar disorder, work disability, social disability, subtype of psychopharmacological treatment and social support. For the logistic regression, the Hosmer-Lemeshow goodness-of-fit test showed that the logistic model was appropriate (χ^2^ = 5.95; df = 8; P = 0.65). Bold value indicates significance level inferior to 0.01*.

**Table 5:** Logistic regression of factors associated with an early age of onset (≤ 20 years) in a bipolar disorder sample (*n* = 108).

**Variables**	***n***	**%**	**Crude OR**	**95% CI**	**Pearson** **χ^2^**	**df**	***P* value**	**Adjusted OR**	**95% CI**	**Wald** **χ^2^**	**df**	***P* value**
Current use of antipsychotics					8.26	1	**0.004**			11.43	1	**0.001**
No	11/60	18	1.0					1.0				
Yes	21/48	44	3.5	(1.4–8.2)				59.8	(5.6–641.9)			
History of psychotic symptoms					1.89	1	0.17			6.03	1	**0.014**
No	17/68	25	1.0					1.0				
Yes	15/40	37	1.8	(0.77–4.2)				13.4	(1.7–106.5)			
	**Mean**	**SD**		**Student's** ***t***	**df**	***P*** **value**	**Adjusted OR**	**95% CI**	**Wald** **χ^2^**	**df**	***P*** **value**	
Age (in years)				4.68	106	** <0.001**	0.72	(0.61–0.84)	16.5	1	** <0.001**	
Patients with early onset	39.2	13.1										
Patients with late onset	52.0	12.8										
Diagnosis delay (in years)				−2.52	40.1	**0.016**	1.5	(1.7–1.9)	17.57	1	** <0.001**	
Patients with early onset	13.0	12.3										
Patients with late onset	7.1	7.2										
Social support score				−0.10	106	0.92	1.1	(1.01–1.2)	4.1	1	**0.042**	
Patients with early onset	34.8	10.0										
Patients with late onset	34.6	10.4										

*In addition to the variables showed in the table, the variables initially included in the logistic regression backward selection procedure were educational level, subtype of bipolar disorder, work disability, family disability and social disability, family history of psychiatric illness, predominant polarity, polarity of the first episode and Clinical Global Impression score, alcohol misuse, nicotine dependence, ever hospitalization and suicide attempts. For the logistic regression, the Hosmer-Lemeshow goodness-of-fit test showed that the logistic model was appropriate (χ^2^ = 3.94; df = 8; P = 0.86). Bold value indicates significance level inferior to 0.01*.

## Discussion

In the present study, we measured the prevalence and the associated variables of PP, of polarity of the onset episode and of early (vs. late) age at onset.

For the PP, we selected a less restrictive definition according to other studies (27, 43), allowing us to determine the polarity in the whole sample, obtaining a distribution of 29.6% for manic PP (32 out of 108) compared to 70.4% for depressive PP (76 out of 108). However, other authors have used a more restrictive definition of the PP (26). No consensus on what is the best option has been reached to date. A consensus is needed because the percentage of patients in which the PP can be determined based on each definition ranges between 42.4 and 71.8%, reaching even 100% for the less restrictive definition (8). Similarly, the distribution of manic PP and depressive PP in the literature ranges from 12 to 55% and from 17 to 34%, respectively (8). We selected a less restrictive definition because our sample size was small, which might explain the high percentage of patients with depressive PP, and such an open definition was needed to increase the statistical power. Therefore, close definitions with criteria that are more difficult to meet are recommended for larger samples; probably these criteria would reflect better the practical reality, where some cases show no clear predominance of one or the other polarity (indeterminate PP), which can represent up to 30% of the total of BD patients (44).

Regarding the association of PP with clinical variables, we find that patients with depressive PP show higher frequency of work disability, a worse clinical condition according to the CGI severity score and more frequent onset by a depressive episode. Consistency between PP and polarity at the onset episode is one of the most replicated findings in the literature (22–24, 28) and our results confirmed the association between depressive PP and depressive polarity at the first episode (the association between manic PP and manic first episode did not reach statistical significance in our study). According to our findings, the polarity of the first episode can become a key factor in the early stages of BD because it may predict later PP in a high percentage of cases, contributing to establish a suitable clinical path from the beginning. Our data on more frequent work disability and worse scores on the CGI severity scale are also consistent with the literature reporting a higher severity in depressive PP due to its more frequent association with variables of poor prognosis, such as comorbidity with anxiety disorders (29) or more frequent and more serious relapses (27). However, in contrast with previous studies, we found no association between depressive PP and other relevant variables, such as high indexes of suicidal behavior (22, 24, 26, 45) or more delay until the right diagnosis of BD (22, 28). These inconsistencies could be explained by differences in methodology and designs of the studies; for example, different criterion to define PP or prospective rather than retrospective design.

The second diagnostic specifier that we analyzed was polarity of the first episode. We found that depressive onset was notably the quite high in our sample (76 out of 108 patients, 70.4%), in line with other studies reporting onset with a depressive episode in two thirds of BD patients (27, 46, 47), although this proportion can be different if mixed episodes are considered (48). In consistency with previous studies (47, 48), we found that depressive onset was associated with type II BD, being one of the factors contributing to a worse perceived quality of life in these patients compared to patients diagnosed with type I BD (49).

One of the most novel findings in our study (not previously reported) is the relation found between depressive onset and both diagnosis delay and family disability, two variables indicating poor prognosis. These two variables affect negatively the clinical course and would strengthen the idea that the long-term clinical course of patients with depressive PP would be worse compared to patients with manic PP (50). Again, some authors found other associations between depressive onset and variables of poor prognosis that we could not replicate, such as higher rates of suicidal behavior (51) or more frequent and lasting relapses (27, 51).

Besides, we aimed to analyze the role of age at onset, since several studies suggest its role as a prognosis variable for BD (9, 52, 53). We decided to split age at onset into early (≤ 20 years) and late (>20 years), based on the criterion used by other authors (53), obtaining among 29.6% (32 out of 108) of the participants an early age at onset, slightly superior to the 22% described by Belteczki et al. (53). Indeed, we found a more frequent association with variables of poor prognosis in these patients; in particular, higher frequency of ever psychotic symptoms and use of antipsychotic drugs, worse social support and higher diagnostic delay, in consistency with the findings described by other authors (52, 53). These results are in line with the most recent meta-analysis on age at onset (9), according to which the risk of poor prognosis increases when the onset of symptoms has been early. However, it remains to be clarified whether the variables found have a direct effect *per se* or if these variables are related with higher suicidal behavior (21) or more mixed symptoms (17) found in these patients. Reaching a consensus among researchers must be a fundamental issue when studying age at onset and its role as a DS in BD (9).

Finally, we should mention the findings of a recently published 5-year prospective study (54), which develops the idea of time ill, according to which not only the number of episodes is important but also the total time that each patient remains in each one of the phases of the disease would determine the prognosis. The mentioned study showed that patients with manic PP spend more time in the manic phase than depressive PP and undetermined PP patients, but it is worth noting that manic PP patients also remained euthymic for longer periods and, therefore, compensated. These are additional data supporting the better evolution of manic PP compared to depressive PP patients.

Among the strengths of our study, we would like want to point out that since it is an observational study, the characteristics of the sample closely resemble that of patients in the usual clinical practice and that we find associations that had not previously been described, such as the association of depressive onset with diagnostic delay and high family disability. All the participants were evaluated by the same clinical researcher and accurately fulfilled the diagnostic criteria of BD. Among the limitations, we should mention the cross-sectional design itself, the relatively small sample size and the selection of an open definition for the PP, which can be less stable over time and, therefore, it can influence the long-term stability of the findings.

In conclusion, PP, polarity of the first episode and age at onset can be good complementary to the current diagnostic criteria for BD because of both their usefulness for the classification and their prognostic impact. All these three characteristics may be differential in each patient and may contribute to focus the psychoeducation and the clinical path on the most important aspects of each patient from the beginning of the disorder, allowing a much more specific approach (8). Certainly, the use of PP, polarity of the first episode and age at onset have some limitations, such as the lack of consensus in their definitions or which features show the patients of undetermined PP. However, the results of the present and previous studies are important and continue representing a promising line of research in which follow-up studies sharing a common methodology are needed.

## Data Availability Statement

The raw data supporting the conclusions of this article will be made available by the authors, without undue reservation.

## Ethics Statement

The studies involving human participants were reviewed and approved by the protocol was approved by the Ethics Committee of the Jaén Hospital Complex. The patients/participants provided their written informed consent to participate in this study.

## Author Contributions

LG-R has participated in the design of the study, collected the data, participated in the statistical analysis, the interpretation of the data and the drafting of the article. JG-J, PG-D, SJ-F, and MC have participated in the interpretation of the data and the drafting of the article. MG has participated in the design of the study, the statistical analysis, the interpretation of the data and the drafting of the article. All authors contributed to the article and approved the submitted version.

## Conflict of Interest

The authors declare that the research was conducted in the absence of any commercial or financial relationships that could be construed as a potential conflict of interest.
